# NOTCH3 as a modulator of vascular disease: a target in elastin deficiency and arterial pathologies

**DOI:** 10.1172/JCI157007

**Published:** 2022-03-01

**Authors:** Kimberly Malka, Lucy Liaw

**Affiliations:** 1 Maine Medical Partners Vascular Surgery and; 2 Maine Medical Center Research Institute, MaineHealth, Scarborough, Maine, USA.

## Abstract

During blood vessel disease, vascular smooth muscle cell (VSMC) expansion and interaction with the matrix trigger changes in gene expression and phenotype. In this issue of the *JCI*, Dave et al. discover a signaling network that drives VSMC expansion and vascular obstruction caused by elastin insufficiency. Using a combination of gene-targeted mice, tissues and cells from patients with Williams-Beuren syndrome, and targeting of elastin in human VSMCs, the authors identified VSMC-derived NOTCH3 signaling as a critical mediator of aortic hypermuscularization and loss of vascular patency. NOTCH3-specific therapies or therapies that target downstream molecular pathways may provide opportunities to minimize VSMC growth and treat cardiovascular disease with minimal side effects.

## The extracellular matrix

Vascular smooth muscle cells (VSMCs) derive from multiple progenitor cell populations during development (1, 2), which may contribute to the diversity of molecular and cellular phenotypes that arise during vascular pathology. Alterations in VSMC proliferation, differentiation, and the acquisition of alternative functional states occur in several diseases. Pathologies related to expanded medial thickness exhibit as hypertension, formations of neointimal lesions manifest as atherosclerosis and other forms of vascular stenosis, and dilation/thinning of the vascular wall results in aneurysms. Recent studies have highlighted the important interactions of VSMCs with the vascular matrix, including elastin, which structurally organizes the medial layer of the blood vessel and is impaired in several human vascular pathologies.

Elastin is a major component of the extracellular matrix and it, along with collagen, makes up a majority of the blood vessel wall. In humans, supravalvular aortic stenosis (SVAS) has been linked to mutations in the elastin gene (*ELN*). SVAS is characterized by nonatherosclerotic arterial wall thickening resulting in ascending aortic stenosis as well as stenosis in pulmonary and coronary arteries (3). In addition to the pulmonary artery stenosis seen in elastin deficiencies in humans, elastin deficiency has also been linked to smaller pulmonary arteries and pulmonary hypertension in a mouse model (4). Although elastin deficiencies do not lead directly to atherosclerosis, degradation of elastin may allow lipid infiltration into atherosclerotic plaques, leading to the progression of atherosclerosis (5). Additionally, multiple studies have investigated the use of tropoelastin and elastin-like recombinamers to combat neointimal hyperplasia in vascular grafts and stents (6, 7).

## NOTCH signaling

NOTCH receptors, as well as their ligands, are involved in cell-fate determination and are known to regulate apoptosis, proliferation, and differentiation. Classical studies of NOTCH signaling in vivo defined its critical roles during vascular development and morphogenesis, and extensive studies have been performed in mouse models and in cell culture (reviewed in refs. 8, 9). NOTCH3 is involved in VSMC growth and differentiation and shows altered expression as a result of vascular injury. In adult VSMCs, NOTCH3 is widely expressed and is the predominant form, although human VSMCs also produce NOTCH2. For example, atherosclerotic plaques from human carotid and femoral arteries typically have abundant NOTCH3 protein that overlaps with and is more widely expressed than contractile markers, such as smooth muscle myosin heavy chain, while a smaller population of VSMCs express NOTCH2 (10). By contrast, NOTCH1 protein in human atherosclerosis seems to be limited to vascular endothelial cells (10).

Not surprisingly, NOTCH mutations are implicated in a number of human diseases, with some affecting the cardiovascular system. A mutation in JAGGED1 (JAG1), a human NOTCH signaling ligand, causes Alagille syndrome. Among other disease manifestations, patients with Alagille syndrome can exhibit structural cardiac abnormalities as well as vascular anomalies, especially ones involving the pulmonary and intracranial arteries (11). Mutations in NOTCH3 are known to cause cerebral autosomal dominant arteriopathy with subcortical infarcts and leukoencephalopathy (CADASIL), which is the most common cause of heritable stroke and vascular dementia in adults (12). On a molecular level, this disease is characterized by wall thickening and morphological changes in VSMCs in the cerebral arteries (12). Increased proliferation and reduced apoptosis of human endothelial cells in pulmonary artery hypertension has been linked to NOTCH1 and NOTCH2 dysregulation (13). Additionally, NOTCH1 mutations are linked to bicuspid aortic valve disease and it is suggested that dysfunctional NOTCH signaling contributes to aortopathies by influencing VSMC differentiation and apoptosis (14).

## Linking elastin deficiency with NOTCH signaling and disease

In this issue of the *JCI*, Dave et al. (15) explore the role of NOTCH signaling in vascular pathology induced by elastin deficiency. The authors demonstrated that decreased elastin suppressed DNA methyltransferase 1 (DNMT1) expression, resulting in decreased DNA methylation in γ-secretase subunit genes, which reduced gene silencing. Higher expression levels of these proteins, including PSEN1 and PSEN2, were associated with increased generation of the active intracellular domain of NOTCH3. Notably, increased NOTCH signaling resulted in aortic hypermuscularization and decreased lumen size and pharmacological inhibition of the γ-secretase complex reversed this phenotype. In VSMC populations, Dave et al. targeted NOTCH3 signaling using Cre driven by smooth muscle α actin 2 (*Acta2*) regulatory sequences. They showed that JAG1 expression in *Acta2*-expressing lineages and cells was required for the elastin insufficiency phenotype, and global loss of *Notch3* also reversed the occlusive phenotype seen in *Eln^–/–^* mice. It has been well described that both elastin and the NOTCH signaling pathway can play a role in cardiac and vascular diseases, and this study demonstrates a link between the two proteins. In general, aortopathies, valvular heart disease, atherosclerosis, and intimal hyperplasia are largely treated by surgical means. Identifying potential therapeutic targets may lead to nonsurgical options for these pathologies as well as other cardiac and vascular diseases.

**Figure 1:**
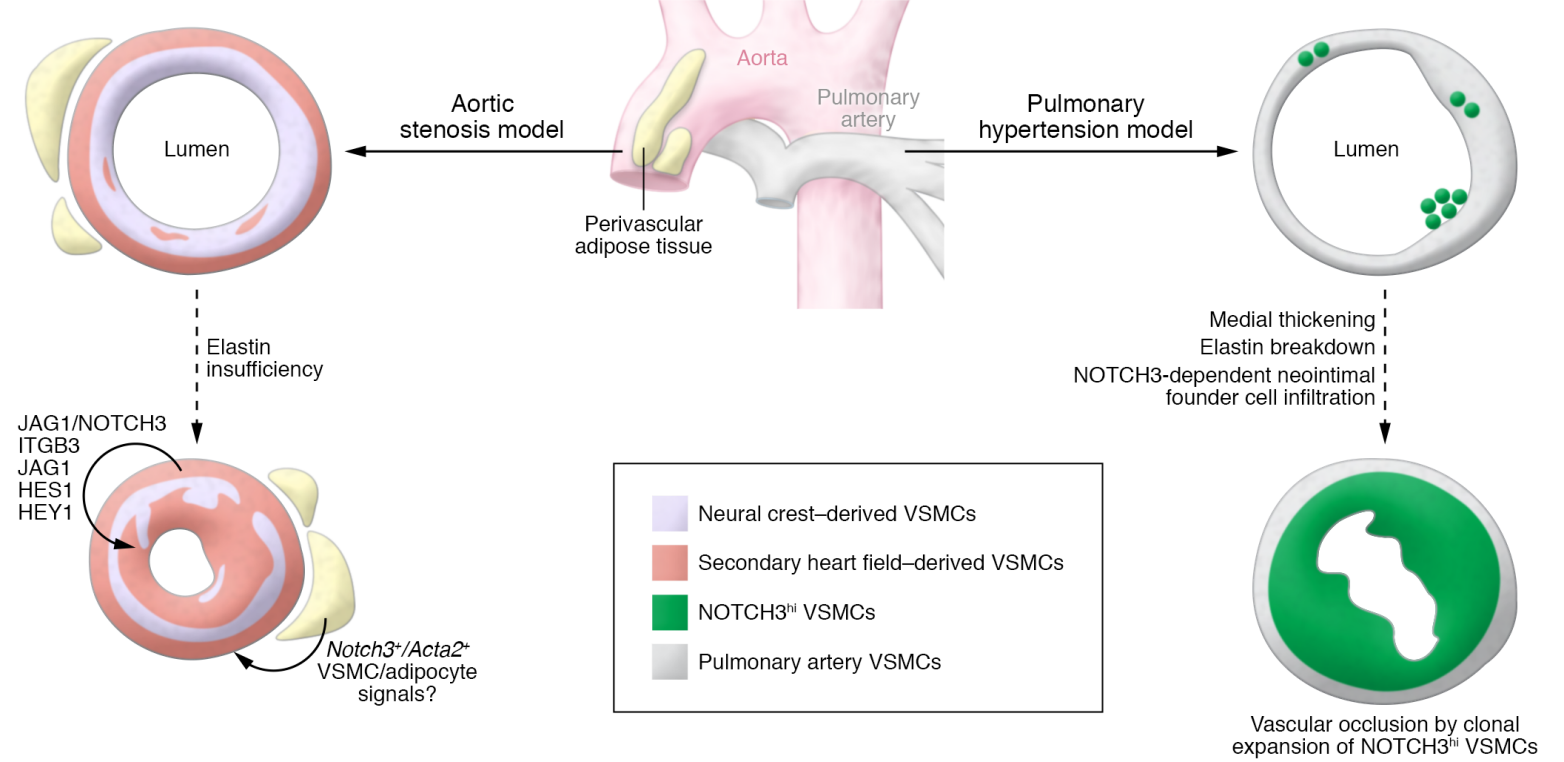
Integration of NOTCH3 signaling mechanisms in vascular occlusive models with elastin insufficiency or elastin degradation. Dave et al. discovered that JAG1/NOTCH3 signaling in VSMCs was critical for neointimal lesion formation with elastin insufficiency. The mechanism involved changes in DNA methylation, NOTCH3 activation through JAG1, and NOTCH targets including integrin β3 (left). Because cells similar to NOTCH3-expressing SMCs reside in the vascular microenvironment within periaortic fat (16), VSMC-specific loss of JAG1, or global loss of NOTCH3, may affect local adipose-derived paracrine signaling (curved arrows). Recently, Lin et al. depleted elastin in a lineage-specific manner to show that VSMCs derived from the secondary heart field lineage drive neointimal lesion formation (23). VSMCs within pulmonary arteries derive from a separate developmental lineage compared with those within the ascending aorta, and, interestingly, NOTCH3 expression varies, with only a small proportion of pulmonary artery VSMCs having high levels (right). Notably, a critical role was discovered for NOTCH3 in a model of chronic pulmonary inflammation leading to medial thickening, elastin breakdown, and intimal lesion formation (24). After elastin breakdown, founder cells required NOTCH3 to infiltrate and populate the intima, leading to clonal expansion and vascular occlusion (24). It will be of interest to query similar roles for NOTCH3 signaling in other pathologies involving VSMC expansion and intimal lesion formation.

Within the vascular microenvironment, VSMCs express NOTCH3. NOTCH3 has also been identified in mouse and human periaortic adipose tissue within a unique population of progenitor cells that share VSMC and adipocyte markers (16). Recent research highlights the ability of local perivascular adipose tissue surrounding the heart and vessels to influence vascular disease, including influencing vascular obstructive disease (17). Given the fact that adipose tissue also forms a rich matrix that includes elastin (18), it is interesting to consider whether changes in this adipose depot within the vascular microenvironment may also impact vascular disease. In the Dave et al. study, presumably the perivascular adipose tissue would also lack elastin and result in altered NOTCH3 signaling in the SMCs and adipocytes. In contrast, VSMCs from pulmonary arteries derive from a different developmental lineage than that of the ascending aorta, with few pulmonary artery VSMCs expressing high NOTCH3 levels. The role of NOTCH3 in the vascular adipose depot, the effects on paracrine signals to the vessel wall, and the roles for NOTCH3 signaling in pathologies related to VSMC expansion in the pulmonary artery require further investigation (Figure 1).

Although NOTCH targeting in cardiovascular disease has not been pursued, inhibition of NOTCH signaling has been studied in other disease processes. γ-Secretase inhibitors were first investigated in Alzheimer’s disease but were found to have undesirable side effects related to widespread inhibition of NOTCH signaling (19). Therefore, these drugs are now being studied in malignancies with known NOTCH involvement. Since γ-secretase inhibitors affect all NOTCH proteins, as well as more than 90 other substrates, one can imagine that there are multiple side effects associated with this family of therapeutics. The main side effect is diarrhea resulting from intestinal toxicity; however, fatigue, rash, cough, nausea, and anorexia have also been reported (20).

As NOTCH is involved in many different diseases, targeting it is very appealing for therapeutic purposes. Designing drugs to target specific NOTCH proteins may provide therapeutic benefit with less of a side-effect profile. Fewer side effects is the idea behind using monoclonal antibodies that target NOTCH receptors or ligands. Many of these antibodies have shown promise in preclinical models and oftentimes have reduced side-effect profiles. One NOTCH3 antibody–drug conjugate (PF-06650808) benefited patients with NOTCH3-positive breast cancer tumors (21). Other NOTCH-targeting therapeutics undergoing clinical trials include γ-secretase modulators, synthetic chemicals, molecular decoys, SERCA inhibitors (which have also been shown to inhibit NOTCH1), modulators of NOTCH glycosylation, and microRNAs (20).

## Conclusions and clinical implications

Therapeutic agents that would treat cardiovascular disease as an alternative to surgery would revolutionize disease management. It seems as though selective inhibition of NOTCH3 combined with a therapeutic that specifically targets VSMCs may provide benefit without the side-effect profile seen with other NOTCH inhibitors. Currently, drugs such as paclitaxel and tacrolimus are delivered locally to the blood vessel wall on angioplasty balloons or intravascular stents as a means to prevent restenosis. It is possible other therapeutic agents could be delivered this way to combat atherosclerosis and intimal hyperplasia. Diseases such as CADASIL and SVAS would require a different approach. Current investigations include using antibodies, hormones, ligands, and other high-affinity molecules that are coupled to carrier molecules, such as liposomes, to deliver drugs to vascular endothelial cells (22). Coupled treatments may be useful in targeting systemic vascular diseases that result from mutations in elastin or NOTCH.

Dave et al. establish an important link between elastin and NOTCH that provides mechanistic information on how these molecules result in many cardiovascular disease states. In developing potential therapeutics, specific NOTCH targeting may be useful in diseases linked to not only NOTCH mutations, but elastin mutations as well. NOTCH3-specific drugs combined with targeted drug delivery may revolutionize the treatment of cardiovascular disease with minimal side effects, and warrant further investigation.

## Version 1 03/01/2022

Electronic publication
